# Systemic and transcriptional response to intermittent fasting and fasting-mimicking diet in mice

**DOI:** 10.1186/s12915-024-02061-2

**Published:** 2024-11-20

**Authors:** Helene Michenthaler, Kalina Duszka, Isabel Reinisch, Markus Galhuber, Elisabeth Moyschewitz, Sarah Stryeck, Tobias Madl, Andreas Prokesch, Jelena Krstic

**Affiliations:** 1 Division of Cell Biology, Histology and Embryology, Gottfried Schatz Research Centre, Medical University of Graz, Graz, Austria; 2 Department of Nutritional Sciences, University of Vienna, Vienna, Austria; 3 Institute of Food, Nutrition and Health, ETH Zürich, Zurich, Switzerland; 4 Institute of Biochemistry, University of Innsbruck, Innsbruck, Austria; 5 Research Centre Pharmaceutical Engineering, Graz University of Technology, Graz, Austria; 6 Division of Medicinal Chemistry, Otto Loewi Research Centre, Medical University of Graz, Graz, Austria; 7 BioTechMed-Graz, Graz, Austria

**Keywords:** Intermittent fasting, Fasting-mimicking diet, Gene expression, Transcription, RNAseq, Mice, Metabolites, Systemic response

## Abstract

**Background:**

Dietary restriction (DR) has multiple beneficial effects on health and longevity and can also improve the efficacy of certain therapies. Diets used to instigate DR are diverse and the corresponding response is not uniformly measured. We compared the systemic and liver-specific transcriptional response to intermittent fasting (IF) and commercially available fasting-mimicking diet (FMD) after short- and long-term use in C57BL/6 J mice.

**Results:**

We show that neither DR regimen causes observable adverse effects in mice. The weight loss was limited to 20% and was quickly compensated during refeeding days. The slightly higher weight loss upon FMD versus IF correlated with stronger fasting response assessed by lower glucose levels and higher ketone body, free fatty acids and especially FGF21 concentrations in blood. RNA sequencing demonstrated similar transcriptional programs in the liver after both regimens, with PPARα signalling as top enriched pathway, while on individual gene level FMD more potently increased gluconeogenesis-related, and PPARα and p53 target gene expression compared to IF. Repeated IF induced similar transcriptional responses as acute IF. However, repeated cycles of FMD resulted in blunted expression of genes involved in ketogenesis and fatty acid oxidation.

**Conclusions:**

Short-term FMD causes more pronounced changes in blood parameters and slightly higher weight loss than IF, while both activate similar pathways (particularly PPARα signalling) in the liver. On individual gene level FMD induces a stronger transcriptional response, whereas cyclic application blunts transcriptional upregulation of fatty acid oxidation and ketogenesis only in FMD. Hence, our comparative characterization of IF and FMD protocols renders both as effective DR regimens and serves as resource in the fasting research field.

**Supplementary Information:**

The online version contains supplementary material available at 10.1186/s12915-024-02061-2.

## Background

During fasting, peripheral organs cooperate to maintain systemic metabolic homeostasis with the major aim of providing the brain with appropriate energy substrates [1, 2]. In modern societies, fasting periods are constantly curtailed due to an over-abundance of high-caloric foodstuffs. Together with our sedentary lifestyle, this often leads to a host of metabolic diseases, such as obesity and diabetes [3, 4]. Dietary restriction (DR), accomplished through a variety of regimens, such as intermittent fasting (IF), time-restricted feeding or different calorie-restricted diets, are potent strategies that have been shown to increase health span and longevity in model organisms from bacteria to non-human primates [5–10]. In general, health benefits of DR arise not only from weight loss and amelioration of metabolic disease [11–14] but also through improvements of the immune [15, 16] and cognitive functions [15, 17].

To circumvent micronutrient deficits during water only fasting and to improve patient compliance, a commercially available diet that mimics the effects of fasting was developed [15, 18]. When fed to mice, fasting-mimicking diet (FMD) provides 50% of the average caloric intake on the first day and 10% of the average caloric intake on the following days. The number of days during which the mice will receive 10% of the average caloric intake can be adapted to animals’ response. For instance, refeeding can be initiated when mice lose approximately 20% of their starting weight. The beneficial effects of FMD have been shown in several disease contexts, such as beta-cell regeneration in the pancreas [19], increasing therapy efficacy in breast cancer [20], or aiding regeneration and fighting autoimmunity in multiple sclerosis [16] or inflammatory bowel syndrome [21]. Intriguingly, in some mouse studies, IF or periodic fasting inflicted lasting effects that were maintained even when interrupted by longer periods of normal nutrient supply [22–24]. Similarly, in a clinical study testing FMD cycles, several of the improved health risk parameters were retained 3 months after the final FMD cycle [25].

Even though fasting and FMD have been used as DR strategies in various studies, the systemic effects of the two have not been directly compared thus far. Hence, we aimed to compare the systemic and transcriptional changes induced by two regimens in mice, defining metabolic effects that can aid the design of future mouse studies in the fasting research field.

We previously showed that fasting causes time-resolved transcriptional changes in the liver, the central organ in the fasting response [26], as well as that both IF and FMD alleviate therapy resistance in hepatocellular carcinoma [27]. Here, we compared the systemic response to fasting and FMD in mice after single and repeated regimens. We also compare the respective transcriptional response in the liver. Our results demonstrate that both DR regimens trigger the anticipated systemic response, with short-term FMD leading to more pronounced changes than IF in systemic parameters and slightly greater weight loss. At the transcriptional level, both DR interventions produced similar pathway-level transcriptional programs, with the PPARα signalling pathway being the most enriched in the liver. However, a more pronounced systemic response was also partly reflected in transcriptional changes upon FMD vs IF. Repeated IF induced similar transcriptional responses as acute IF, while repeated cycles of FMD resulted in blunted expression of genes involved in ketogenesis and fatty acid oxidation.

## Results

### Intermittent fasting and FMD in mice

**Fig. 1:**
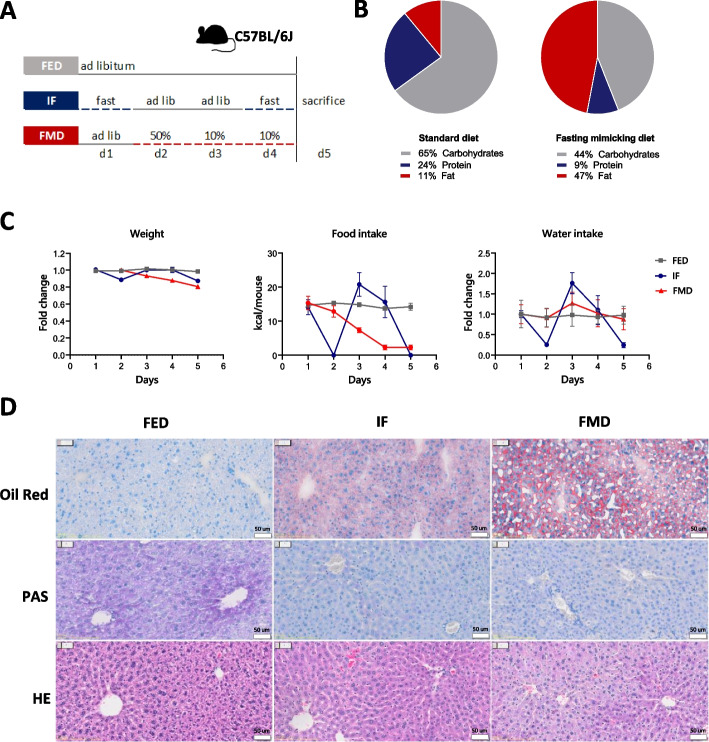
Intermittent fasting and fasting-mimicking diet in mice. **A** Experimental design: C57BL/6 J mice were either fed standard diet ad libitum (FED, *n* = 6), intermittently fasted (IF, *n* = 6) for 24 h on days 1 and 4, or fed a fasting-mimicking diet (FMD, *n* = 8) on days 2–4. Animals were sacrificed on day 5. **B** Macronutrient composition of the standard diet and FMD. **C** Body weight, food and water intake during the 5 days of the experiment. **D** Representative Oil Red O, PAS and H&E staining of the livers from fed, IF and FMD-fed mice

To compare the systemic response to short-term IF and FMD, we employed these two regimens in the C57BL/6 J mouse strain (Fig. 1A). In the control group, mice had ad libitum access to the standard diet containing 65% carbohydrates, 24% protein and 11% fat (Fig. 1B). In the IF group, mice were food-deprived two times for 24 h with two ad libitum refeeding days in between (Fig. 1A). The third group was fed FMD containing 44% carbohydrates, 9% protein and 47% fat (Fig. 1B). On the first day, mice received FMD containing 50% of their average calorie intake, and on the second and third day they received 10% of their average calorie intake (Fig. 1A).

During fasting in the IF group, mice lost on average 17% of their starting body weight, which was regained upon refeeding. The mice fed FMD lost weight gradually, reaching an average 20% weight loss after day 3 (Fig. 1C). On refeeding days, the mice in the IF group increased their food intake by approximately 10% compared to standard chow diet-fed mice (Fig. 1C). As water intake was reduced in the IF group during food restriction (Fig. 1C), mice in IF and FMD group were provided ad libitum access to hydrogel to avoid dehydration. Furthermore, transient liver steatosis typically occurring upon fasting was observed in both fasted and FMD-fed mice, with higher lipid accumulation in the FMD group (Fig. 1D). Both IF and FMD resulted in glycogen depletion in the liver (Fig. 1D).

### Systemic metabolite changes after acute DR

**Fig. 2:**
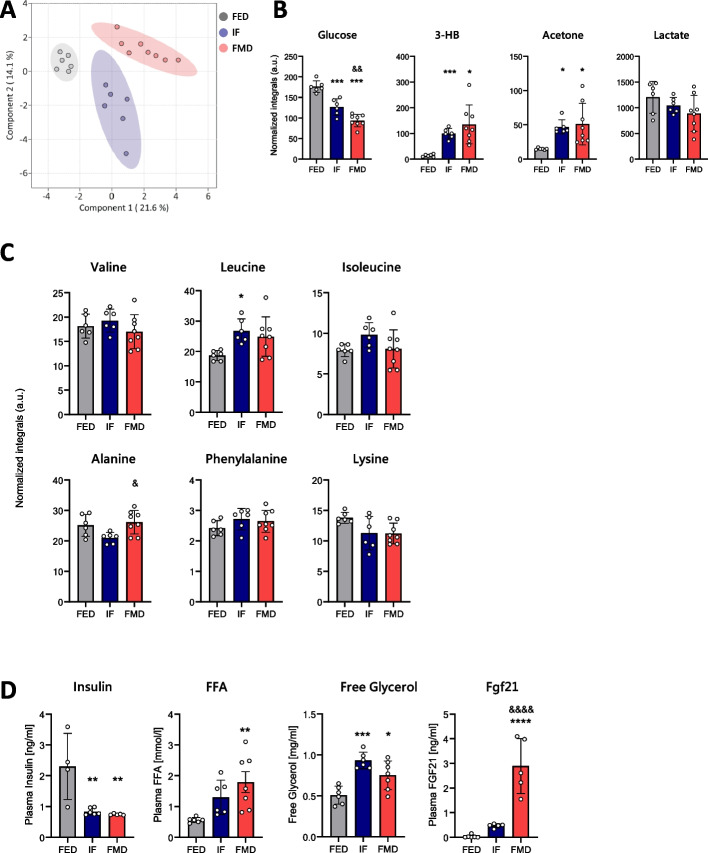
Systemic metabolite changes after acute dietary restriction. Plasma was collected on day 5 of the experiment. **A** sPLS-DA plot of NMR data of plasma samples from mice fed standard diet (FED, *n* = 6), intermittently fasted (IF, *n* = 6), or fed fasting-mimicking diet (FMD, *n* = 8). **B** Glucose, 3-HB, acetone and lactate concentration in the plasma of mice. **C** Branched-chain amino acid concentrations in the plasma of mice. **D** Insulin, free fatty acid (FFA), glycerol and FGF21 levels in the plasma of mice. Sample sizes (*n*) for each group: insulin (FED *n* = 4, IF *n* = 6, FMD *n* = 5); FFA (FED *n* = 6, IF *n* = 6, FMD *n* = 7); glycerol (FED *n* = 6, IF *n* = 6, FMD *n* = 7); FGF21 (FED *n* = 6, IF *n* = 5, FMD *n* = 5). Data are presented as mean values ± SEM. Significant differences were analysed by two-way ANOVA with Bonferroni post hoc tests. *p* < 0.05 (*), *p* < 0.01 (**), *p* < 0.001 (***); *IF/FMD vs FED, ^&^IF vs FMD. Individual data values can be found in Additional file 7: Table 3

We next analysed blood plasma of mice upon IF and FMD by nuclear magnetic resonance (NMR)-based metabolomics. Sparse partial least squares discriminant analysis (sPLS-DA) based on the metabolite composition revealed distinct clustering of the three groups (Fig. 2A). Both IF and FMD induced expected systemic response in mice. Glucose concentration was significantly reduced after IF, with even stronger decrease in the plasma of FMD-fed mice (Fig. 2B). Furthermore, ketone bodies (3-hydroxybutyrate (HB) and acetone) were significantly increased in plasma of both IF and FMD-fed mice, while lactate was slightly reduced in both groups (Fig. 2B). Moderate changes in plasma amino acid content were observed, with branched-chain amino acids (BCAA) more abundant in the IF group when compared to standard diet or FMD group. In addition, alanine levels were significantly reduced in the IF group when compared to FMD group, and no statistically significant difference was observed in phenylalanine and lysine levels (Fig. 2C).

As expected, insulin levels were significantly reduced, while the concentration of free fatty acids (FFA) and free glycerol were significantly increased in plasma after both IF and FMD (Fig. 2D). Interestingly, we observed more than three times higher increase in FGF21 concentration in the plasma of FMD-fed vs IF mice (Fig. 2D).

### Genome-wide transcriptional regulation in response to acute DR

**Fig. 3:**
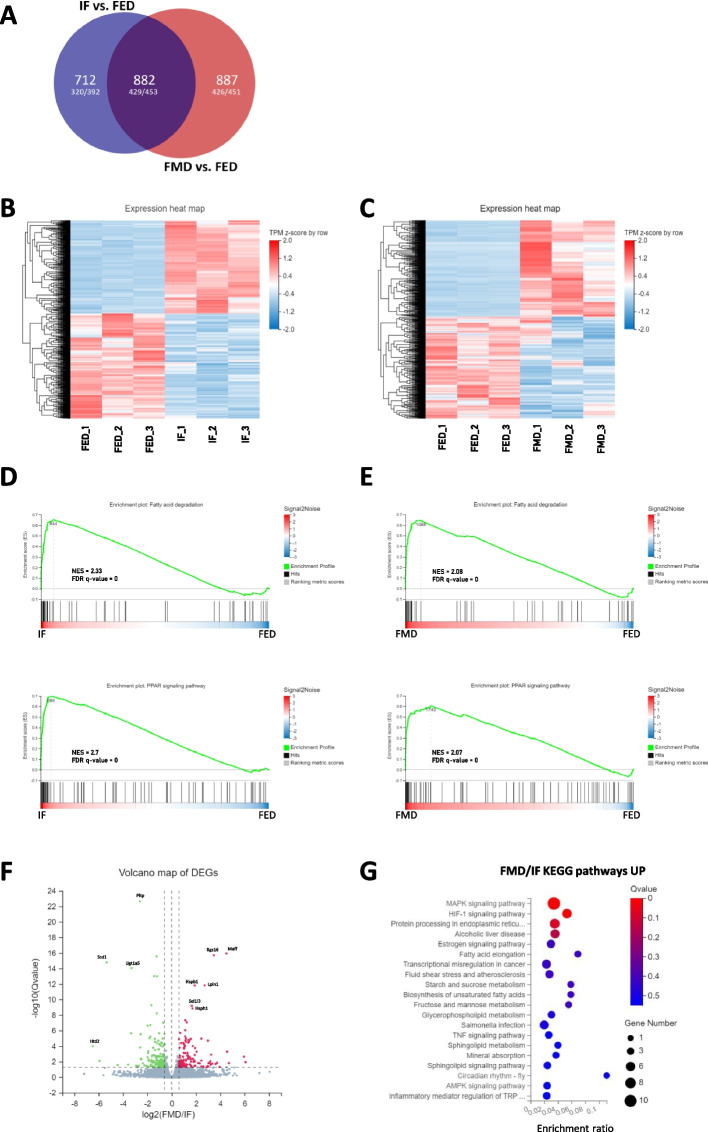
Genome-wide transcriptional reprogramming in response to acute dietary restriction. **A** Venn diagram illustrating the number of differentially expressed genes that are common and distinct in FMD-fed and IF mice; **B** Heatmap of differentially expressed genes (DEGs) in the liver of IF mice vs the mice fed standard diet (749 genes upregulated and 845 genes downregulated) as well as **C** mice fed FMD vs mice fed standard diet (855 upregulated genes and 904 downregulated genes) with upregulated transcripts shown in red and downregulated transcripts shown in blue. Values are presented as transcripts per million (TPMs); false discovery rate (FDR) 5%. DEGs were identified by comparing expression levels of each gene between groups; **D** Gene Set Enrichment Analysis (GSEA) for “KEGG fatty acid degradation” and “KEGG PPAR signalling pathway” in IF mice compared to mice fed standard diet as well as **E** mice fed FMD compared to mice fed standard diet. Dr.Tom analysis tool (BGI) was used to map GSEA hallmark gene sets (NES, normalized enrichment score); **F** Volcano plot showing DEGs with a fold change ≥ 1 and FDR 5% between IF and FMD groups; **G** KEGG pathway analysis of genes differentially upregulated in IF compared to FMD-fed mice. X-axis shows the enrichment factor and the y-axis indicates the associated KEGG pathway. The size of the dots reflects the number of differentially expressed genes associated with each pathway, and the colour of the dots indicates the *q* values

To investigate the transcriptome changes underlying the metabolic response to acute fasting, we carried out RNA sequencing (RNA-seq). A large number of genes were detected as uniquely deregulated in either DR regimen when compared to the fed group. While a total of 882 differentially expressed genes were common to IF and FMD as compared to fed group (Fig. 3A), 712 and 877 genes were uniquely differentially expressed compared to fed group in IF and FMD, respectively (Fig. 3A). Hierarchical cluster analysis of gene expression patterns further confirmed distinct transcriptional profiles for IF and FMD each compared to the fed group (Fig. 3B and C). KEGG pathway analysis of differentially expressed genes (DEGs) between IF and fed group demonstrated that upregulated DEGs were significantly enriched in multiple metabolic pathways, with PPAR signalling, fatty acid degradation and metabolism, as well as p53 signalling pathway ranking as top enriched pathways (Additional file 1: Fig. S1). Pathways related to protein processing and steroid biosynthesis were predominantly downregulated (Additional file 1: Fig. S1), reflecting a shift from anabolic to catabolic metabolism during fasting regardless of the DR regimen. Despite many uniquely IF or FMD differentially regulated genes (Fig. 3A), on pathway level a comparable transcriptional program was observed in FMD group compared to fed group (Additional file 2: Fig. S2). Gene Set Enrichment Analysis (GSEA) also revealed strong enrichment in pathways related to fatty acid degradation and PPAR signalling in both the IF and FMD group when compared to the fed group (Fig. 3D and E). This suggests that both DR interventions induce similar metabolic adaptations, particularly with respect to lipid metabolism (Fig. 3B and C).

Examination of key genes in metabolic KEGG pathways revealed transcriptional changes in both IF and FMD groups compared to the fed control (Additional file 1: Fig. S1C and Additional file 2: Fig. S2C). Strong changes in gene expression were observed across several pathways, including glycolysis/gluconeogenesis, PPAR signalling, p53 signalling and lipid metabolism for both DR interventions. With the chosen cut-off, the IF group showed a higher number of significantly changed genes within the investigated pathways compared to FMD-fed group (Additional file 1: Fig. S1C and Additional file 2: Fig. S2C).

Based on these observations, differential effects between the two DR interventions were analysed. A volcano plot of DEGs with a logFC of 0.585 and a *p* value < 0.05 highlighted the most significantly up- and downregulated genes between IF and FMD (Fig. 3F). For example, we found Hspb1 (heat shock protein family B member 1) and lipin 1 (Lpin1) to be higher expressed in the liver of FMD-fed mice, while the expression of stearoyl-CoA desaturase 1 (Scd1) was more pronounced upon IF (Fig. 3F).

KEGG pathway analysis demonstrated that FMD-upregulated DEGs were significantly enriched in mitogen-activated protein kinase (MAPK) signalling, hypoxia-inducible factor 1 (HIF-1) signalling and fatty acid elongation (Fig. 3G). Hence, direct comparison of IF and FMD responses suggests that while IF and FMD groups share common features in their transcriptional response, they also induce distinct signalling and metabolic pathways that may contribute to unique physiological effects.

### Transcriptional regulation of the metabolic response to acute DR

**Fig. 4:**
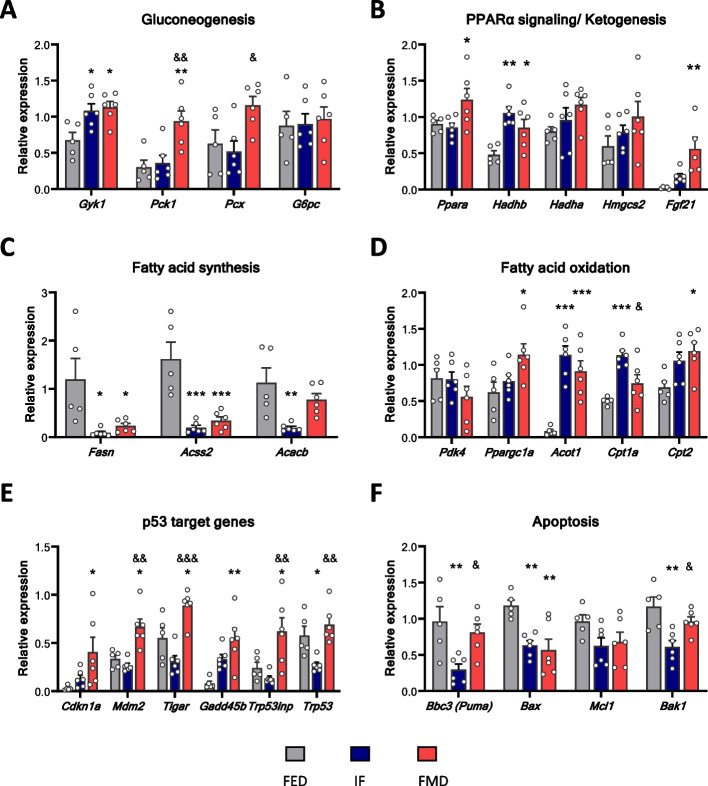
Transcriptional regulation of the metabolic response to acute dietary restriction. Relative mRNA expression of **A** gluconeogenesis-relevant genes, **B** PPARα signalling/ketogenesis-relevant genes, **C** fatty acid synthesis-relevant genes, **D** fatty acid oxidation-relevant genes, **E** p53 target genes and **F** apoptosis-relevant genes in the liver of FED (*n* = 5), IF (*n* = 6) and FMD (*n* = 6) mice. Significant differences were analysed by two-way ANOVA with Bonferroni post hoc tests. *p* < 0.05 (*), *p* < 0.01 (**), *p* < 0.001 (***); *IF/FMD vs FED, ^&^IF vs FMD. Individual data values can be found in Additional file 8: Table 4

Building on our RNA-seq data, we validated the expression of hepatic metabolic pathway-related genes in all three groups using qPCR. The main functions of the analysed genes are described in Additional file 3: Table 1. The expression of *Pck1* and *Pcx* genes involved in gluconeogenesis was significantly changed only after FMD, while IF did not lead to significant differences in the expression of any of the genes, except *Gyk1* (Fig. 4A). Strikingly and in line with the RNAseq data, *Ppara* and *Fgf21* expression was significantly increased only after FMD (Fig. 4B), Fgf21 expression reflecting its increased concentration in blood (Fig. 2D). When compared to the fed group, significant increase in the expression of *Hadhb*, and a trend of increased expression of additional ketogenesis-involved genes (*Hadha* and *Hmgsc2*) was observed, however without significant difference between the IF and FMD groups (Fig. 4B). Genes coding for enzymes needed for fatty acid (FA) synthesis (*Fasn*, *Acss2* and *Acacb*) underwent potent downregulation after both IF and FMD (Fig. 4C). In contrast, some of the genes coding FA oxidation enzymes (*Ppargc1a*, *Acot1*, *Cpt1a*, *Cpt2*) were strongly upregulated in both IF and FMD groups, indicating accelerated FA oxidation under ketogenic conditions (Fig. 4D). As we previously published an involvement of p53 in response to acute fasting [28, 29], we also measured the mRNA expression of *Trp53* and its target genes. *Trp53* expression levels increased after FMD and were reduced after IF. In contrast, downstream target genes of p53 were significantly upregulated after FMD. However, no significant differences were detected after acute IF compared to control (Fig. 4E). Genes coding for apoptosis-related proteins showed a trend of decreased expression in both groups when compared to control (Fig. 4F). In line, TUNEL staining of liver sections did not show substantial elevation in apoptotic cell content in IF and FMD groups compared to the fed group (Additional file 4: Fig. S3).

### Systemic metabolite changes after repeated IF and refeeding

**Fig. 5:**
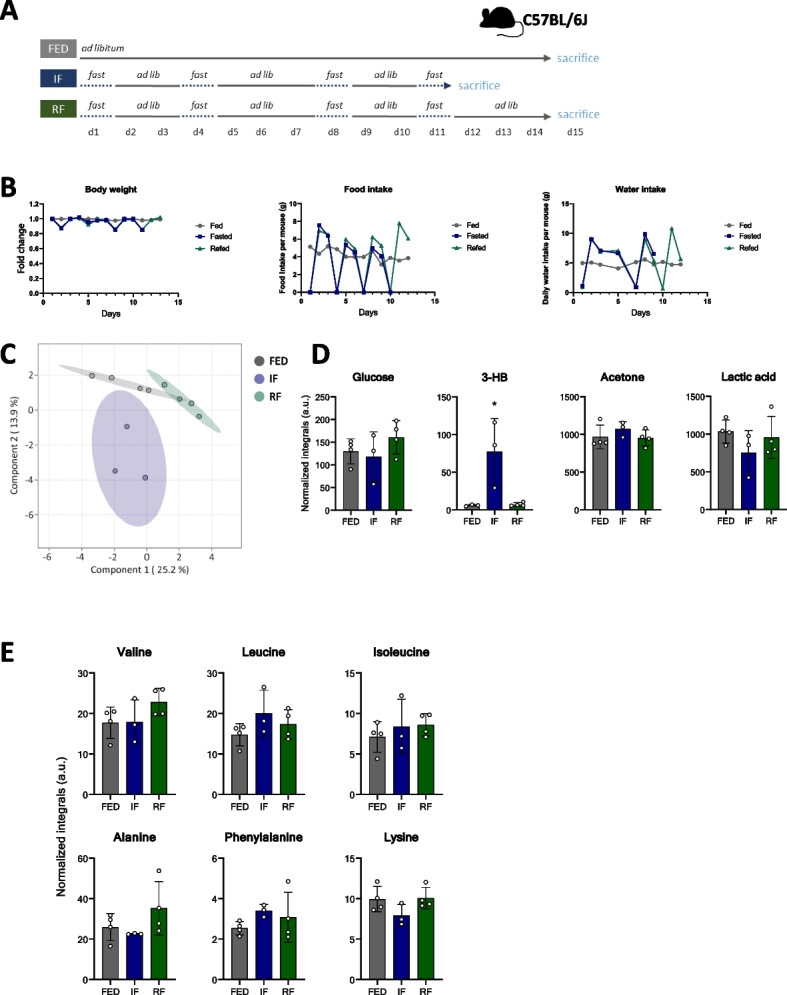
Systemic metabolite changes after repeated intermittent fasting and refeeding. **A** Experimental design: C57BL/6 J mice were either fed a standard diet and sacrificed on day 15 (FED, *n* = 4), intermittently fasted (IF, *n* = 3) and sacrificed on day 12, immediately after fasting, or on day 15 after re-feeding (RF, *n* = 4). **B** Body weight, food and water intake during the experiment. **C** Plasma was collected after the last day of the experiment, before the mice were sacrificed. sPLS-DA analysis of the NMR data. **D** Glucose, 3-HB, acetone and lactate concentration in the plasma of mice. **E** Branched-chain amino acid concentrations in the plasma of mice. Significant differences were analysed by two-way ANOVA with Bonferroni post hoc tests. *p* < 0.05 (*), *p* < 0.01 (**), *p* < 0.001 (***); *IF/FMD vs FED, ^&^IF vs FMD. Individual data values can be found in Additional file 9: Table 5

To determine the effects of repeated IF and the dynamics of their reversibility, mice were fasted for 24 h four times with 2–3 intermittent refeeding days, with one group sacrificed immediately after the fourth fasting day and another after 3 days of refeeding (Fig. 5A). The control group was fed ad libitum (Fig. 5A). On fasting days, mice lost 8–15% of their starting body weight. Weight was completely regained during refeeding (Fig. 5B). In the IF group, food intake was higher than in control group, especially on the first refeeding day (Fig. 5B). Similar to acute fasting (Fig. 1C), water intake followed the pattern of food intake (Fig. 5B). Metabolome data was clearly conditionally separated (Fig. 5C) and changes in plasma metabolite concentrations after four fasting cycles (Fig. 5D and E) were less pronounced when compared to those observed after two cycles (Fig. 2B and C). After 3 days of refeeding, most of the metabolites returned to their previous concentrations (Fig. 5D). Transient liver steatosis and glycogen depletion upon IF were also reversed after refeeding (Additional file 5: Fig. S4).

### Transcriptional regulation of metabolic pathways after repeated IF and refeeding

**Fig. 6:**
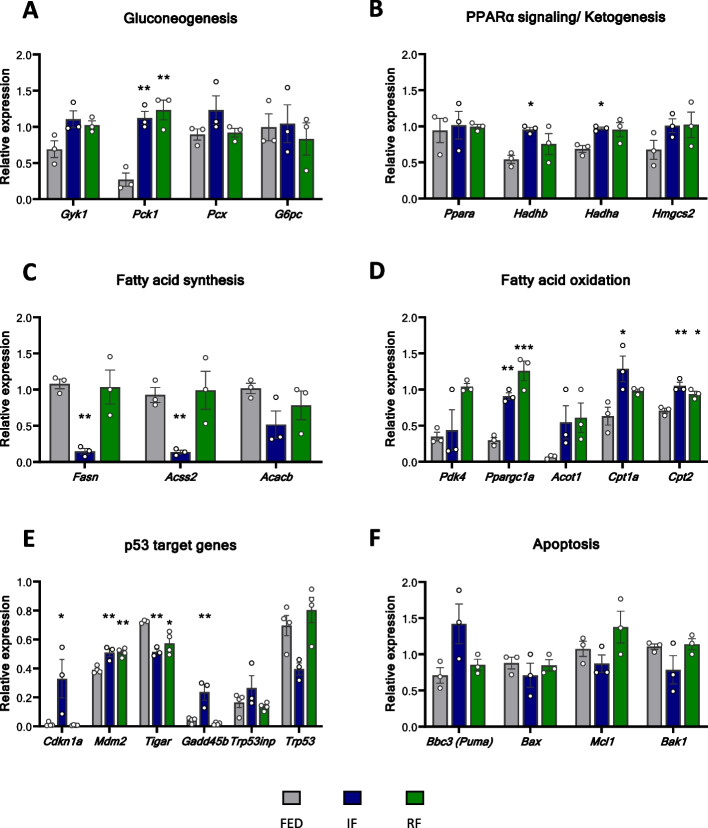
Transcriptional regulation of metabolic response after repeated intermittent fasting and refeeding. Relative mRNA expression of **A** gluconeogenesis-relevant genes, **B** PPARα signalling/ketogenesis-relevant genes, **C** fatty acid synthesis-relevant genes, **D** fatty acid oxidation-relevant genes, **E** p53 target genes and **F** apoptosis-relevant genes in the liver (*n* = 4 mice per group). Significant differences were analysed by two-way ANOVA with Bonferroni post hoc tests. *p* < 0.05 (*), *p* < 0.01 (**), *p* < 0.001 (***); *IF/FMD vs FED, ^&^IF vs FMD. Individual data values can be found in Additional file 10: Table 6

While most genes coding for gluconeogenic enzymes showed only a trend towards upregulation after repeated fasting, the expression of *Pck1* was significantly increased after repeated IF and this increased expression remained high after 3 days of refeeding (Fig. 6A). Most genes coding for ketogenic enzymes were induced upon repeated fasting and remained high after refeeding (Fig. 6B). After strong downregulation upon fasting, the genes coding for enzymes involved in FA synthesis returned to normal expression levels upon refeeding (Fig. 6C). Intriguingly, the genes coding for enzymes involved in FA oxidation remained upregulated (*Acot1*, *Cpt1a* and *Cpt2*), or were even higher upregulated after refeeding, compared to the levels immediately after fasting (*Pdk4*, *Ppargc1a*) (Fig. 6D). Similarly, the mRNA expression of certain fasting responsive p53-target genes was significantly increased (*Cdkn1a*, *Mdm2* and *Gadd45b*) after repeated IF and the expression levels mostly returned to basal levels after 3 days of refeeding (Fig. 6E). Apoptosis-relevant genes showed no statistically significant difference after repeated fasting, except that *Bbc3* was strongly increased. However, *Bbc3* returned to normal expression levels after refeeding (Fig. 6F).

### Transcriptional regulation of metabolic pathways after repeated cycles of FMD

**Fig. 7:**
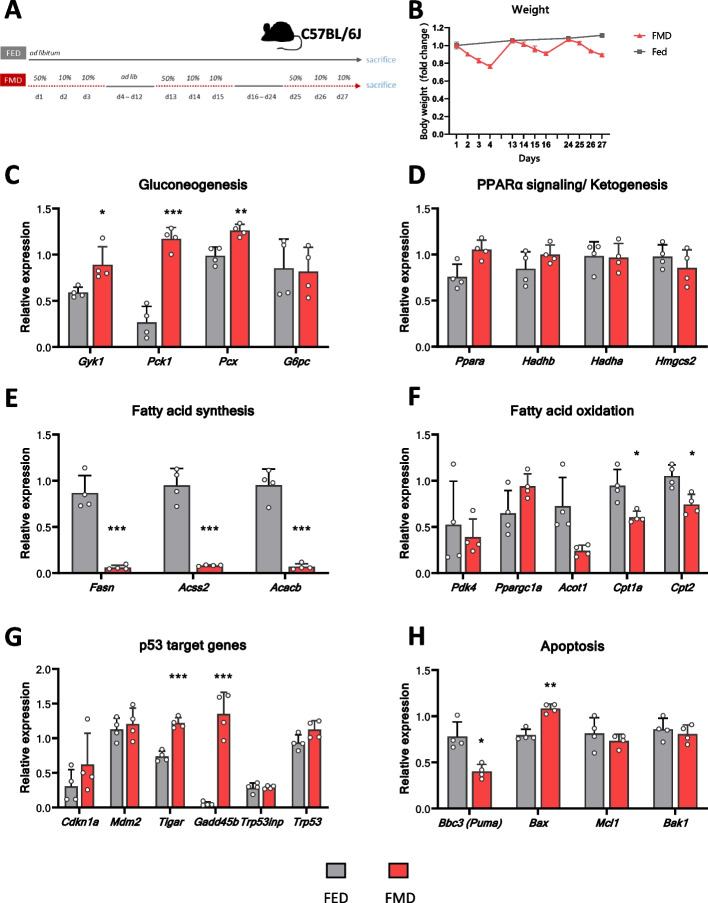
Transcriptional regulation of metabolic genes after repeated cycles of fasting-mimicking diet. **A** Experimental design: C57BL/6 J mice were either fed a standard diet (FED, *n* = 4) or fed three cycles of fasting-mimicking diet (FMD, *n* = 4) and sacrificed on day 28. **B** Body weight during the experiment. Relative mRNA expression in the liver: **C** gluconeogenesis-relevant genes; **D** PPARα signalling/ketogenesis-relevant genes; **E** fatty acid synthesis-relevant genes; **F** fatty acid oxidation-relevant genes; **G** p53 target genes; **H** apoptosis-relevant genes. Significant differences were analysed by two-way ANOVA with Bonferroni post hoc tests. *p* < 0.05 (*), *p* < 0.01 (**), *p* < 0.001 (***); *IF/FMD vs FED, ^&^IF vs FMD. Individual data values can be found in Additional file 11: Table 7

To investigate the feasibility and fasting response upon repeated cycles of FMD, mice received three cycles of FMD for 3 days (50%-10%-10% average calorie intake) with eight refeeding days in between. The control group had ad libitum access to the standard chow diet (Fig. 7A). Again, mice fed FMD lost weight gradually, reaching an average 20% weight loss after 3 days on diet; during refeeding weight was completely regained (Fig. 7B).

The expression of genes involved in gluconeogenesis was significantly increased after repeated cycles of FMD diet compared to fed group (Fig. 7C), while the expression of ketogenesis-involved genes was not changed (Fig. 7D). Consistent with the strongly reduced expression after one FMD cycle (Fig. 4C), the expression of genes coding for enzymes involved in FA synthesis was blunted after repeated cycles of FMD (Fig. 7E), which was similar to repeated IF (Fig. 6C). Some genes coding for FA oxidation enzymes were significantly downregulated (*Acot1*, *Cpt1a*, *Cpt2*) in the FMD group indicating depleted fat sources after multiple cycles of FMD (Fig. 7F). Some p53 target genes (*TIGAR*, *Gadd45b*) were significantly increased after repeated cycles of FMD (Fig. 7G). Genes coding for apoptosis tended to be reduced upon FMD, except for *Bax* which was significantly increased (Fig. 7H).

## Discussion

Dietary restriction is the most potent intervention known to increase health and lifespan in preclinical animal models, as well as to reduce ageing-related disease markers in humans [30, 31]. Many modalities of DR have been developed over the years, with a common goal to make the diets more applicable and to increase diet adherence in patients [6]. One such modality is the FMD, first published in 2015 [15]. Both fasting and FMD represent non-pharmacological interventions that show positive effects on several organs at the same time without causing severe adverse effects like most drugs. However, we still lack a deeper understanding of the exact molecular mechanisms and acute and long-term effects of fasting regimens on the cellular and tissue level. Although fasting and FMD are already relatively well investigated, the direct comparison of their systemic implications is still missing.

In our study, both DR regimens induced a potent fasting response, reflected in decreased glucose and insulin concentrations and increased ketone body, FFA and FGF21 concentrations in blood. Mice fed FMD, in comparison to IF, had lower glucose levels and higher levels of 3-hydroxybutyrate, FFAs and most notably FGF21 in blood, suggesting a stronger acute fasting response after FMD.

On the transcriptome level, despite differences in specific genes, IF and FMD instigated similar transcriptional programs at the pathway level, particularly in lipid metabolism. PPARα signalling pathway was the top enriched pathway in the liver after both IF and FMD, although the expression of the *Ppara* gene itself was only significantly increased after FMD. Liver PPARα is one of the key transcription factors involved in the adaptive response to fasting [32]. It plays a pivotal role in regulating ketogenesis, FGF21 production and overall metabolic homeostasis during nutrient fluctuations [33]. FGF21, a hormone that plays a critical role in energy expenditure, lipid metabolism and glucose homeostasis, acts systemically to further promote fatty acid oxidation, improve insulin sensitivity and modulate feeding behaviour [34]. As mentioned, the concentration of FGF21 in the plasma of FMD-fed mice was more than three times higher in the plasma IF mice. These findings confirm the role of FGF21 as key metabolic regulator induced during fasting that is primarily controlled by PPARα in the liver [35], and also go in line with the overall stronger fasting response to FMD, also reflected in higher FFA concentration in the plasma, and higher expression of *Ppara* in the liver.

In line with our previously published data [26], short-term IF did not cause significant induction in the expression of *Pcx* and *G6pc* genes. However, contrary to previously published data, *Gyk1* and *Pck1* showed a trend towards an increase after acute fasting and a significant increase only after long-term IF or both acute and repeated FMD. This discrepancy, as well as the absence of increased fasting response genes after acute IF, could be caused by different experimental conditions in this and previous study (e.g. grouped housing vs single caging) [26]. In addition, increased expression of p53 target genes after FMD could also be caused by activation of other transcription factors (e.g. Foxo1) that are activated after longer DR regimens such as longer periods of fasting [36]. It is important to note that changes in metabolite levels upon DR do not necessarily reflect the changes in mRNA expression, resulting from variances in the duration of the transcriptional and metabolic end effect changes to occur. In general, the transcriptional changes identified in our study mostly agree with previous studies investigating fasting [26, 37, 38]. Some discrepancies in gene expression were observed and could be due to nuances in experimental design (e.g. duration of DR, sampling time point, mouse strain).

Four cycles of fasting in our study resulted in similar metabolic and transcriptomic changes as two cycles of fasting, similar to what was observed in an earlier study with shorter fasting protocol [37]. Almost all the metabolites and gene parameters reflecting the response after four cycles of fasting returned to standard values after 3-day refeeding. Only some genes involved in gluconeogenesis and FA oxidation were still highly expressed after refeeding, probably due to high, fasting-induced lipid content in the liver. Metabolic flexibility (adaptation to DR/fasting periods) has also been recently reported after repeated cycles of FMD [39]. However, three cycles of FMD led to a decrease in most genes involved in FA synthesis and oxidation. One explanation for this could be that repeated FMD cycles exhausted adipose tissue depots in mice, which would deplete circulating free FAs pre-empting their metabolism in the liver.

Similar effects on gut microbiota and immune status after long-term IF and FMD have recently been reported [40]. This study also demonstrated that FMD and IF induce similar outcomes as calorie restriction and, to a lesser extent, ketogenic diet. In addition to our findings, this confirms that IF and FMD elicit mostly similar systemic responses.

So far, fasting and FMD have been proven safe in clinical applications suggesting they could benefit public health as one promising implementation of preventive medicine [12, 13, 25, 31, 41, 42]. One of the advantages of FMD is its easy application, which allows for higher control of nutritional intake in clinical studies. The diet is separately assembled for daily use, which allows higher flexibility in the protocol duration. However, compliance to multiple cycles of FMD remains an issue [43, 44], even though to a lesser extent than in the case of IF. Clinical trials have proven FMD safe for use in cancer patients [18, 43–47], as well as in healthy individuals [48]. In the recently completed clinical study in cancer patients, the incidence of severe adverse effects was 12.9% in patients receiving FMD, while only 4% of the patients presented with severe hypoglycaemia [43, 46]. Of note, FMD was combined with anticancer therapy in these studies. In our mouse model, the animals withstood DR and recovered soon after refeeding.

Although fasting constitutes a simple and effective regimen with numerous beneficial effects on health and disease, the patient compliance remains one of the main hurdles in its clinical use, in addition to potential malnutrition and unwanted weight loss or cachexia in patients with cancer. From that perspective, FMD might be more applicable than IF and long-term dietary regimens because it avoids malnutrition through supplementation of essential vitamins and minerals [18] and offers the possibility of adapting the diet to patient-specific real-time systemic changes (e.g. in case of hypoglycaemia the FMD cycle can be shortened from 5 to 3 or 4 days). Furthermore, based on clinical studies, it is advised that FMD should be consumed by humans at most once a month and up to three to four times per year by healthy individuals [18, 31]. Needless to say, consumption of FMD, as any other fasting protocol should be supervised by health practitioners.

It is important to note that the FMD composition used in our mouse experiments is identical to that used in human patients and while the caloric restriction in mice always follows the same pattern (50% on day 1 and 90% on days 2–5), in clinical trials calorie restriction (50–70% on day 1 and 70% on days 2–5), as well as FMD cycle duration and refeeding time, differ depending on the study [49]. Hence, the direct comparison of parameters determining the response to FMD in mice and humans is difficult. Still, the overall induction of a fasting response (transient weight loss coupled with lower blood glucose, insulin and increase in ketone bodies) is observed in all different versions of the FMD protocol. In addition to our findings, a more detailed understanding of the exact molecular mechanisms of DR would help to foster the field of fasting mimetics that could be developed into pharmacotherapies.

## Conclusions

We show that both DR regimens elicit a distinct systemic response, with short-term FMD causing more pronounced changes in systemic parameters and slightly higher weight loss. On transcriptional level, both DR regimens resulted in mostly similar transcriptional programs at the pathway level with PPARα signalling pathway as top enriched pathway in the liver. In addition, FMD increased the expression of genes driving gluconeogenesis and PPARα signalling/ketogenesis more potently than IF. Furthermore, FMD showed higher expression of p53 target genes than IF, while apoptosis, a major downstream pathway of p53, was not significantly affected by both DR. Similar transcriptional response was observed upon acute and repeated IF, while repeated cycles of FMD lead to dampened expression of ketogenesis and fatty acid oxidation-relevant genes.

## Methods

### Mouse experiments

All in vivo experiments were performed in accordance with the European Directive 2010/63/EU and approved by the Austrian Federal Ministry of Education, Science and Research (ref. no. BMWFW-66.010/0160-WF/V/3b/2017). Mice were housed under standard 12-h light/12-h dark cycles.

Animals were randomly assigned to groups and were either ad libitum fed standard diet (Altromin 1320 fortified); intermittently fasted, meaning that the food was withheld for 24 h two times per week (with ad libitum access to water and hydrogel), with 2 or 3 days of ad libitum refeeding in between; or fed FMD, with ad libitum access to water and hydrogel. FMD was purchased from Prolon and was prepared as already published [15]. One FMD cycle lasted 3 days: 1st day accommodated 50% of the average calorie intake and days 2–3 accommodated 10% of the average calorie intake. For the long-term experiment, mice were kept on an ad libitum diet for 9 days between the FMD cycles. The protocol was repeated for three cycles. Average food intake was calculated for all mice based on their standard diet intake 1 week in advance. In the refeeding group, mice were refed standard diet ad libitum for 3 days and then sacrificed by cervical dislocation.

### Plasma parameters analysis

Plasma insulin levels were determined using the Ultra-Sensitive Mouse Insulin ELISA Kit (CrystalChem, Zaandam, Netherlands) according to manufacturer’s instructions. Plasma free fatty acids (FFA) and free glycerol concentrations were measured using NEFA-HR kit (Wako Chemicals GmbH, Germany) and Free Glycerol Reagent (Sigma-Aldrich, St. Louis, USA), respectively, according to manufacturer’s instructions. Plasma FGF21 concentration was measured using an ELISA from BioVendor (BioVendor R&D, Modrice, Czech Republic) according to manufacturer’s instructions.

### Histological staining

Liver tissues were fixed overnight in 4% paraformaldehyde solution and paraffin embedded for sectioning. Standard staining with haematoxylin and eosin was performed on 5 µm sections.

Glycogen was detected with a standardized periodic acid Schiff (PAS) staining technique. Therefore, 5 µm sections were deparaffinized and rehydrated in periodic acid for 25 min at room temperature. After washing in water, sections were incubated in Schiff´s reagent for 25 min, washed with water for 5 min and counterstained with haematoxylin.

Terminal deoxynucleotidyl transferase-mediated dUTP nick end-labelling (TUNEL) staining was performed on paraffin-embedded liver sections after antigen retrieval (93 °C, 7 min at pH 6) according to the manual for In Situ Cell Death Detection Kit (Roche, Basel, Swiss). Positive control was pre-treated with DNase I (3000 U/ml) and for negative control slides were treated with label solution instead of TUNEL reaction mix. All cell nuclei were stained with DAPI, and nuclei with positive signal in FITC channel were considered TUNEL-positive.

Oil Red O staining was performed to detect lipid accumulation in liver tissue. Therefore, frozen Sects. (6 µm) were prepared with a Cryostar, allowed to dry for 30 min at room temperature to avoid detachment and stored in a refrigerator until stained. Sections were rinsed with 60% isopropanol, stained in a freshly prepared Oil Red O working solution (60% of 0.5 g/100 ml Oil Red O stock solution dissolved in 99% isopropanol, 40% distilled water) for 10 min at room temperature. Stained sections were washed in 60% isopropanol and washed by three changes of water. Images were obtained using Olympus VS200 slide scanner.

### RNA isolation from tissue samples

Flash-frozen tissues were lysed in Qiazol reagent and dissociated by using stainless steel beads (Qiagen, Hilden, Germany) at 50 Hz for 1 min (3 runs) in the TissueLyser (Qiagen, Hilden, Germany). In between runs, samples were cooled on ice for 3 min. RNA was isolated using the PeqGOLD total RNA kit (Peqlab, Radnor, USA), quantified via Nanodrop (Thermo Fisher Scientific, Waltham, MA, USA) and stored at − 20 °C.

### qPCR analysis

Isolated RNA was reverse transcribed to cDNA using the RevertAid RT Reverse Transcription Kit (Thermo Fisher Scientific, Waltham, MA, USA), diluted to a final concentration of 1 µg/µl before cDNA was amplified using Blue SybrGreen qPCR Mastermix (Biozym Scientific, Olendorf, Germany). Relative mRNA concentrations were normalized to the expression of TATA-box binding protein (TBP) and transcription factor IIB (TFIIb) and quantified using the comparative ΔCt method.

Primer sequences are listed in Additional file 6: Table 2.

### RNA sequencing (RNA-seq) and data analysis

Total RNA was isolated from liver samples of three mice per group from acute DR cohorts as described above. Library preparation and RNA sequencing were performed by the Beijing Genomics Institute (BGI; Shenzhen, China). Following quality control (RIN > 8), samples underwent oligo-dT enrichment, fragmentation and reverse transcription. Strand-specific mRNA libraries were prepared and sequenced using DNA nanoball technology (DNBSEQ, BGI). Data were analysed using Dr.Tom data visualization solution (BGI) including Kyoto Encyclopedia of Genes and Genomes (KEGG) pathway analysis, Gene Set Enrichment Analysis (GSEA), hierarchical clustering and Venn diagram. Genes with an adjusted *p* value < 0.05 and log2FC > 0.585 were considered differentially expressed.

### NMR sample preparation

For polar metabolite extraction, 140 µl ice-cold methanol was added to 70 µl plasma, tubes were mixed by shaking, and stored at − 20 °C for 1 h. After centrifugation at 13,000 rpm for 30 min (4 °C), the supernatant was transferred to new 1.5 ml tubes and subsequently lyophilized. Plasma metabolic analysis was conducted at 310 K using a Bruker Advance Neo 600 MHz NMR spectrometer equipped with a TXI probe head. The Carr-Purcell-Meiboom-Gill pulse sequence was used to acquire ^1^H one-dimensional NMR spectra with presaturation for water suppression (cpmgpr1d, 512 scans, 73,728 points in F1, 12,019.230 Hz spectral width, recycle delay of 4 s). NMR spectral data were processed as previously described [50]. Shortly, data were processed in Bruker Topspin version 4.0.2 using one-dimensional exponential window multiplication of the FID, Fourier transformation and phase correction. NMR data were then imported to Matlab2014b, TSP was used as an internal standard for chemical shift referencing (set to 0 parts per million), regions around the water, TSP and methanol signals were excluded, NMR spectra were aligned, and a probabilistic quotient normalization was performed. Quantification of metabolites was carried out by signal integration of normalized spectra and use of the TSP signal as internal standard.

### Statistical analyses

Statistical analyses were performed using GraphPad Prism 8 (GraphPad Software). Statistically significant differences were determined as described in the figure legends. If not noted otherwise, data represent mean values ± SEM with the following grades of statistical significance: *p < 0.05, **p < 0.01 and ***p < 0.001.

## Supplementary Information

Additional file 1: Fig. S1 KEGG pathway analysis and differential expression of key metabolic genes based on RNAseq data from the livers of fed and intermittently fasted mice.

Additional file 2: Fig. S2 KEGG pathway analysis and differential expression of key metabolic genes based on RNAseq data from the livers of fed and FMD-fed mice.

Additional file 3: Table 1 Main functions of metabolic genes selected for qPCR analyses.

Additional file 4: Fig. S3 Apoptosis detection in the liver upon acute dietary restriction.

Additional file 5: Fig. S4 Histologic properties of the liver upon acute dietary restriction.

Additional file 6: Table 2 Primer sequences used for qPCR.

Additional file 7: Table 3 Individual data values for Fig. 2

Additional file 8: Table 4 Individual data values for Fig. 4

Additional file 9: Table 5 Individual data values for Fig. 5

Additional file 10: Table 6 Individual data values for Fig. 6

Additional file 11: Table 7 Individual data values for Fig. 7

## Data Availability

All data generated or analysed during this study are included in this published article and its supplementary information, or are available in publicly accessible repositories. NMR measurement data can be found in Additional files of the manuscript. The RNA sequencing data have been deposited in the Gene Expression Omnibus (GEO) repository under accession code GSE279842 (https://www.ncbi.nlm.nih.gov/geo/query/acc.cgi?acc=GSE279842).

## Abbreviations

Acacb: Acetyl-CoA carboxylase beta
Acot1: Acyl-CoA thioesterase 1
Acss2: Acyl-CoA synthetase short chain family member 2
Bax: BCL2-associated X protein
Bak1: BCL2-antagonist/killer 1
Bbc3: BCL2 binding component 3
BCAA: Branched-chain amino acids
Cdkn1a: Cyclin dependent kinase inhibitor 1A
Cpt1a: Carnitine palmitoyltransferase 1a
Cpt2: Carnitine palmitoyltransferase 2
DEGs: Differentially expressed genes
DR: Dietary restriction
FA: Fatty acid
Fasn: Fatty acid synthase
FFA: Free fatty acids
FGF21: Fibroblast growth factor 21
FITC: Fluorescein isothiocyanate
FMD: Fasting-mimicking diet
Gadd45b: Growth arrest and DNA damage inducible beta
GSEA: Gene Set Enrichment Analysis
Gyk1: Glycerol kinase
G6pc: Glucose-6-phosphatase, catalytic
Hadha: Hydroxyacyl-CoA dehydrogenase trifunctional multienzyme complex subunit alpha
Hadhb: Hydroxyacyl-CoA dehydrogenase trifunctional multienzyme complex subunit beta
HIF-1: Hypoxia-inducible factor 1
Hmgcs2: 3-Hydroxy-3-methylglutaryl-Coenzyme A synthase 2
Hspb1: Heat shock protein family B member 1
IF: Intermittent fasting
KEGG: Kyoto Encyclopedia of Genes and Genomes
Lpin1: Lipin 1
MAPK: Mitogen-activated protein kinase
Mcl1: Myeloid cell leukaemia sequence 1
Mdm2: Mouse double minute 2 *homologue*
NMR: Nuclear magnetic resonance
PAS staining: Periodic acid Schiff staining
Pck1: Phosphoenolpyruvate carboxykinase 1
Pcx: Pyruvate carboxylase
Pdk4: Pyruvate dehydrogenase kinase, isoenzyme 4
PPAR: Peroxisome proliferator-activated receptor
Ppara: Peroxisome proliferator activated receptor alpha
Ppargc1a: Peroxisome proliferative activated receptor, gamma, coactivator 1 alpha
RNA-seq: RNA sequencing
Scd1: Stearoyl-CoA desaturase 1
sPLS-DA: Sparse partial least squares discriminant analysis
TBP: TATA-box binding protein
TFIIb: Transcription factor IIB
TIGAR: TP53 induced glycolysis regulatory phosphatase
Trp53: Transformation related protein 53
Trp53inp1: Transformation related protein P53 inducible nuclear protein 1
TUNEL assay: Terminal deoxynucleotidyl transferase-mediated dUTP nick end-labelling
3-HB: 3-Hydroxybutyrate
