# Supplementary figures and images for: Systemic and transcriptional response to intermittent fasting and fasting-mimicking diet in mice
Source: BMC Biol. 2024 Nov 20;22:268. doi: 10.1186/s12915-024-02061-2 (PMC11580389; doi:10.1186/s12915-024-02061-2)

Figure S1

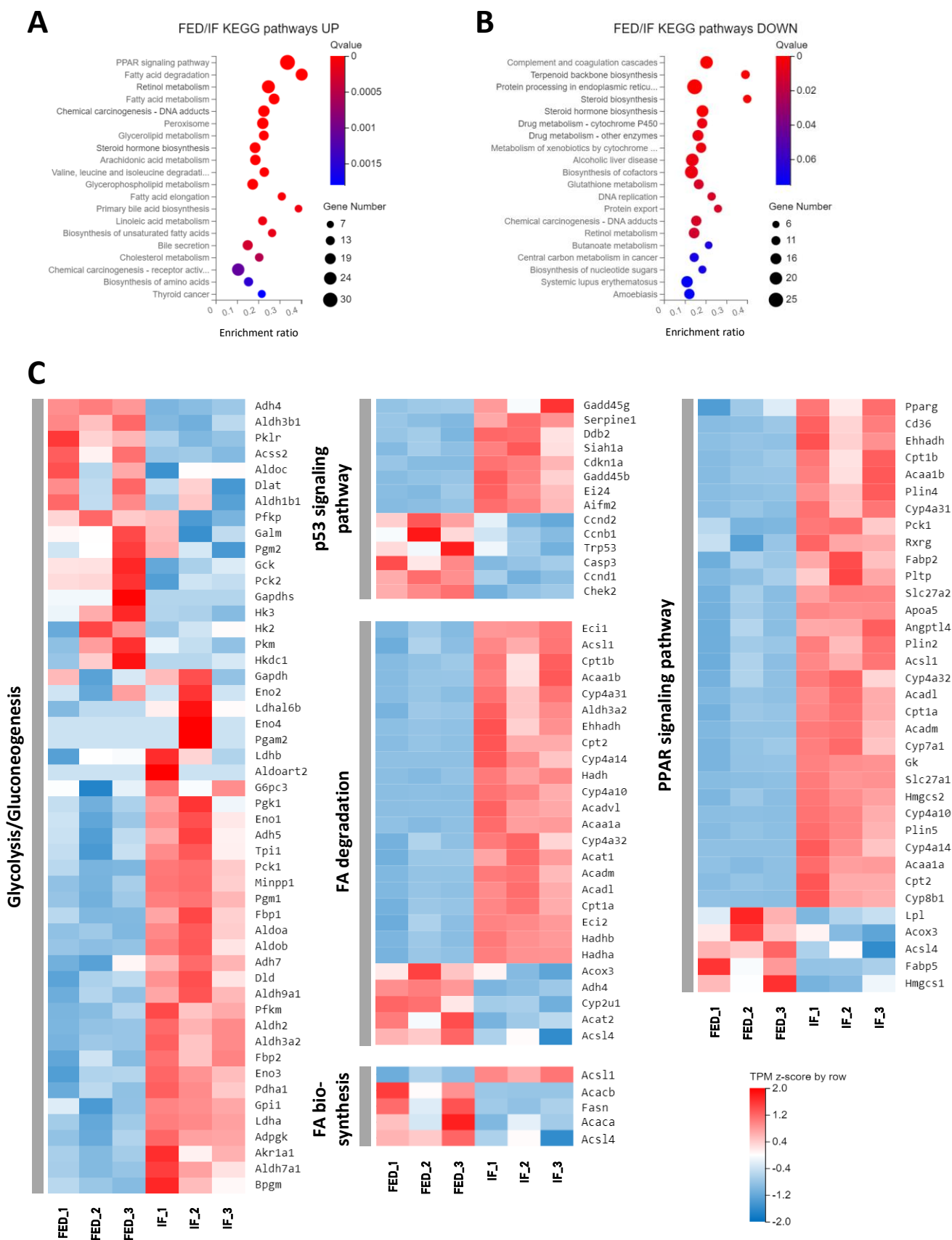

Supplement: Supplementary file 1 — Additional file 1: Fig. S1 KEGG pathway analysis and differential expression of key metabolic genes based on RNAseq data from the livers of fed and intermittently fasted mice. [file 12915_2024_2061_MOESM1_ESM.pdf]

Figure S2

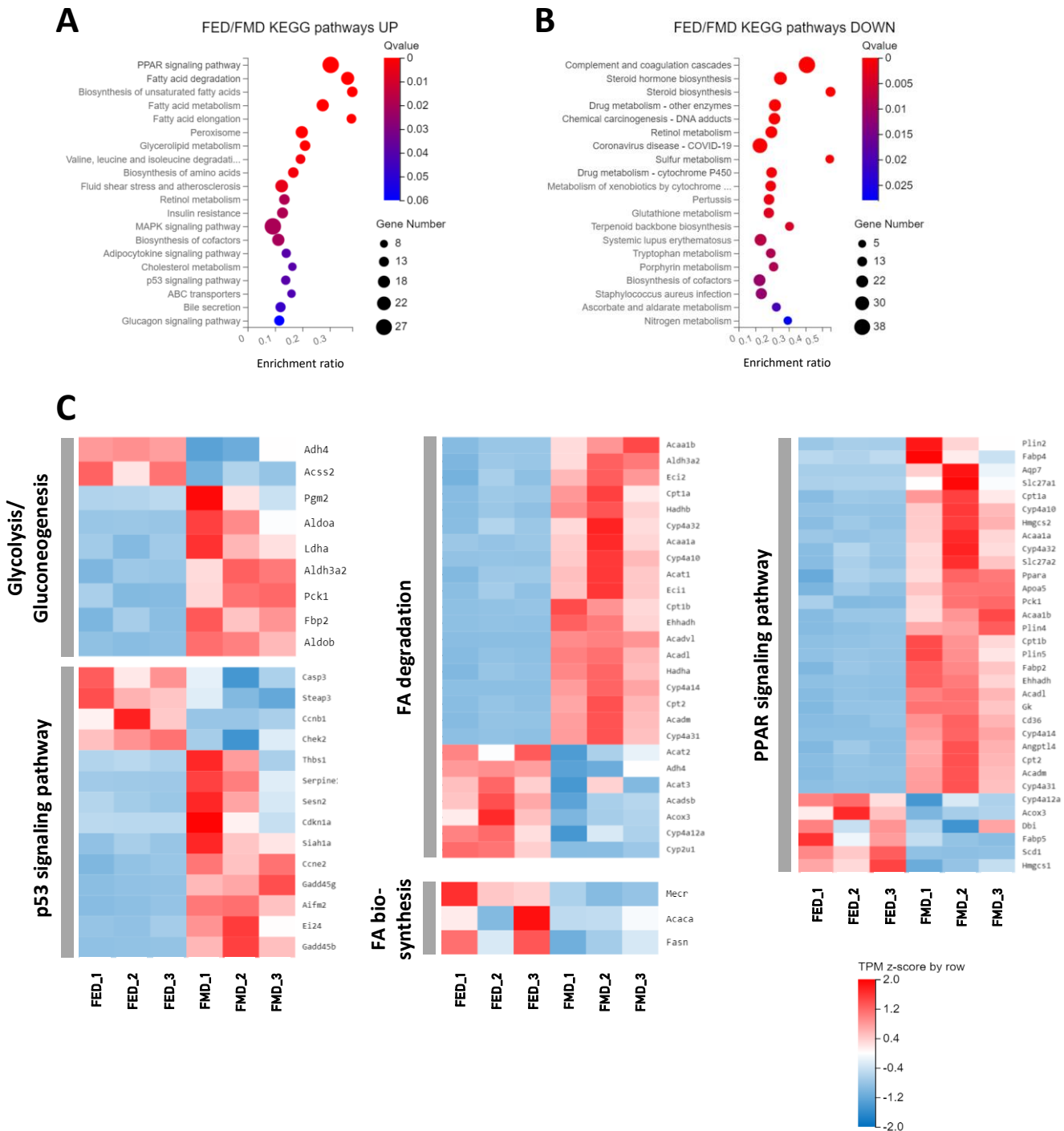

Supplement: Supplementary file 2 — Additional file 2: Fig. S2 KEGG pathway analysis and differential expression of key metabolic genes based on RNAseq data from the livers of fed and FMD-fed mice. [file 12915_2024_2061_MOESM2_ESM.pdf]

Figure S3

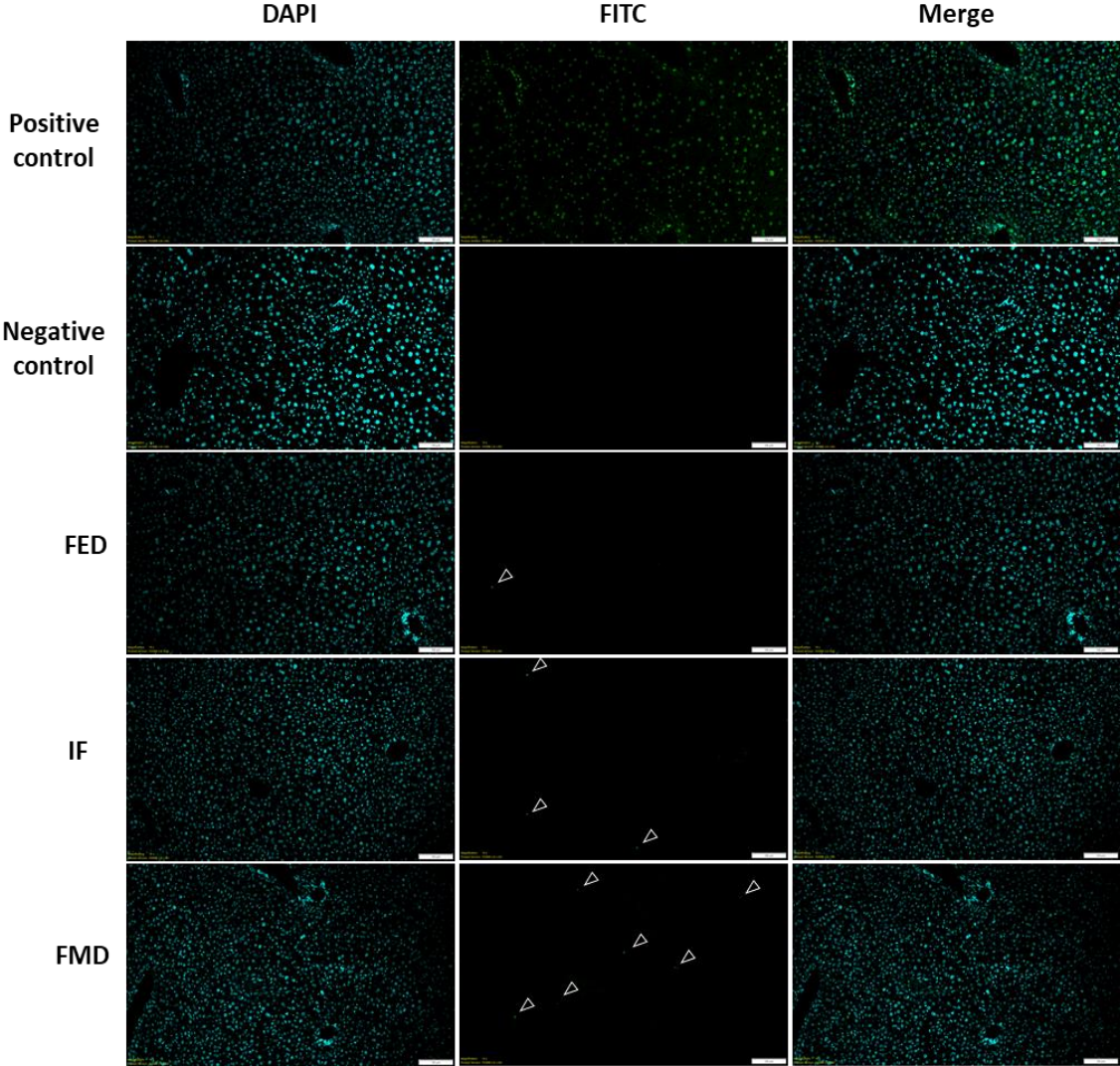

Supplement: Supplementary file 4 — Additional file 4: Fig. S3 Apoptosis detection in the liver upon acute dietary restriction. [file 12915_2024_2061_MOESM4_ESM.pdf]

Figure S4

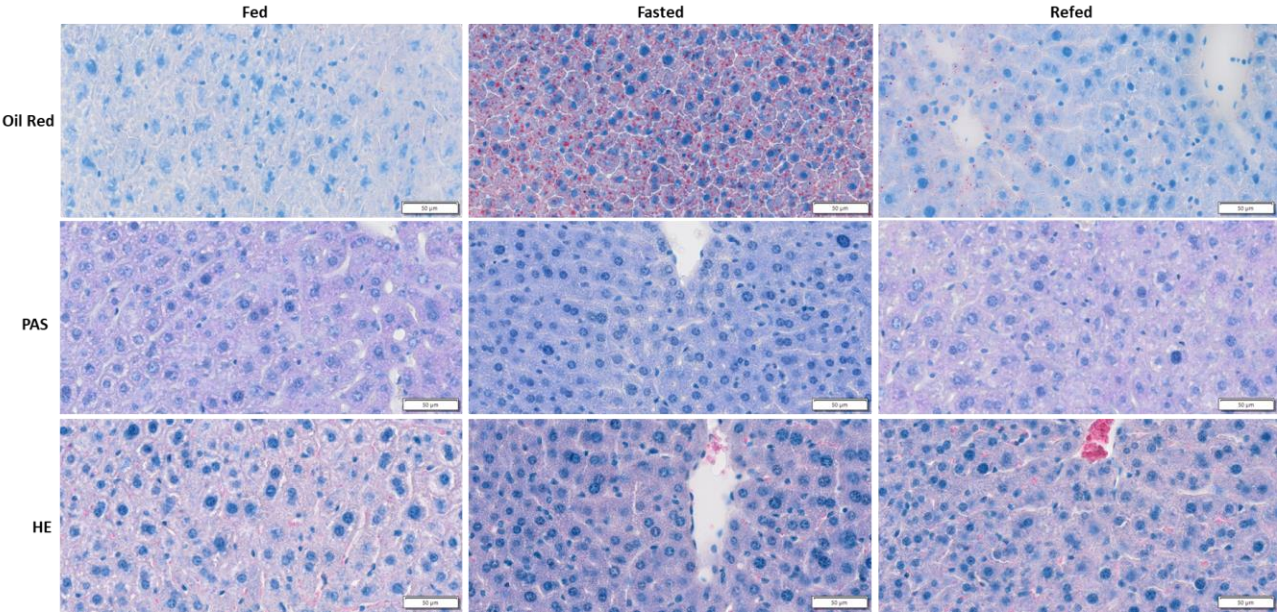

Supplement: Supplementary file 5 — Additional file 5: Fig. S4 Histologic properties of the liver upon acute dietary restriction. [file 12915_2024_2061_MOESM5_ESM.pdf]
